# The First Observational Study of Acute Medical Unit in Qatar

**DOI:** 10.1055/s-0044-1788996

**Published:** 2024-08-22

**Authors:** Abdel-Naser Elzouki, Phool Iqbal, Mohammed Zahid, Ijaz Kamal, Anand Kartha, Mustafa Al-Tekreti, Dabia Al-Muhanadi, Ahmed Al-Mohamed

**Affiliations:** 1 Department of Medicine, Hamad General Hospital, Hamad Medical Corporation, Doha, Qatar; 2 Department of Medicine, College of Medicine, Qatar University, Doha, Qatar; 3 Department of Medicine, Wiell Cornell Medical College-Qatar, Doha, Qatar

**Keywords:** acute medical unit, acute medicine, emergency department, length of stay, postdischarge clinic, readmission

## Abstract

**Background** Acute medical unit (AMU) is a dedicated facility to treat patients with acute medical conditions requiring a short hospital stay (< 72 hours) with the support of a multidisciplinary team led by a medical consultant. We aim to present a study of the AMU model of care from Qatar to provide insight into its effects on patient care and management.

**Methods** Retrospective data from AMU facility at Hamad General Hospital (HGH), Doha, Qatar, was collected from January 2019 to December 2020 from the electronic patient record. The data were analyzed for demographic characteristics of the patients, length of stay (LOS), readmission rate, and postdischarge follow-up. The effectiveness of the AMU system was studied closely from this data. An extensive literature search was also performed for comparative results analysis in other AMU facilities outside Qatar.

**Results** Total admissions under the AMU facility were 8,371 from january2019 to December 2020. The 28 days readmission rate was 10.25 and 9.9% in 2019 and 2020, respectively. The average LOS was approximately 3.2 days. Around 88.7% of the patients were discharged home, 7.8% were admitted to medical wards due to longer stays, and 0.5% left against medical advice. Most of the patients admitted under AMU were 18 to 60 years old. The top primary diagnoses of admissions were minor stroke, transient ischemic attack, chest infection, urinary tract infections, and gastrointestinal and liver diseases. The most common comorbidities were hypertension, diabetes, acute kidney injury, and chronic kidney disease. A total of 2,858 patients were booked for a follow-up visit in the postdischarge clinic on discharge from the AMU for the year 2019 and 2020. The analysis of these followed up patients showed 73% of patients were discharged from clinic after first visit while the readmission from clinic was on only 1% (28 patients for year 2019 and 2020).

**Conclusion** Attentive patient care under AMU with a designated multidisciplinary medical team led by an internal medicine consultant is the cornerstone for the success of the AMU unit. This unit has proven very helpful for the smooth disposition of patients from the emergency department with reduced LOS, readmission rate, and overall mortality.

## Introduction

The acute health care system worldwide is under much pressure, with a decrease in the number of acute care beds and a growth in the number of patients living with multiple health problems and the aging population. 1 On the other hand, the shortage of acute care beds has led to an increasing number of patients boarded in the emergency department (ED) waiting for an inpatient bed. 2 ED crowding with patients is associated with increased morbidity, mortality, and extended hospital stay. 3

Multiple strategies and measures have been implemented globally to improve the flow of patients requiring hospitalization from the ED to the medical floor. The acute medical unit (AMU) facility was introduced in the United Kingdom in 1990 to tackle the bed crisis and overcrowding in ED. It is defined by the Royal College of Physicians of London as “a dedicated facility within a hospital that acts as the focus for acute medical care for patients who have presented as medical emergencies to the hospital.” 4 Later, it was recognized as a separate subspecialty in 2003, with full specialty rights in 2009. 5 The overall proven efficacy of this system has been reported in the literature regarding patient care and reduction in the burden on ED and inpatient medical wards. 4 6 With good results, the AMU facility has already been introduced in other countries like Australia, New Zealand, Italy, Singapore, and Ireland. 6 7 8 Studies from these countries have shown the effectiveness of AMUs in better utilization of hospital resources and improved overall mortality. 6 7

Qatar is multiethnic, and its health care system has been ranked among the top 20 countries in the Healthcare Index by Country 2021. 5 Qatar is also faced with increasing numbers of medical admissions and overcrowding of the ED. The average number of medical admissions per year is more than 10,000 as per Business Intelligence Unit, 2022, Hamad Medical Corporation report. To tackle these challenges, an AMU unit has been established to provide efficient care to specific medical patients requiring acute care on presentation to the ED in 2015 as a part of the General Internal Medicine Department of HGH. Since then, this facility has successfully delivered optimum care to medical patients with the expected discharge within 72 hours of admission to the AMU. The main goal is to provide high-quality care to medical patients with reduced length of stay (LOS), prompt follow-up after discharge, and reduce the burden of ED boarders and inpatient medical wards with improved patient flow.

A dedicated 35-bed AMU was established in June 2015 at HGH, a premier nonprofit health care center in Qatar, to look after acute medical patients with an anticipated LOS of less than 72 hours. The AMU has a medical team led by a consultant and is supported by a multidisciplinary team (MDT) consisting of senior nursing staff, case managers, discharge coordinators, clinical pharmacists, social workers, physiotherapists, and occupational therapists. AMU has its own point-of-care testing facilities, including glucometers, ketometers, urine analyzer, arterial blood gas machine and ultrasound, 4 beds with a central cardiac monitoring facility, and a MDT meeting room. Blood investigations, imaging, and endoscopy requests are prioritized for patients in the AMU wherever possible. The AMU is supported by a purpose-designed ambulatory care area, where discharged patients are followed up when required. Almost all patients are seen within 1 week of discharge unless a later follow-up is desirable.

The admission criteria to the AMU are based on anticipated discharge within 72 hours of clinically and hemodynamically stable patients with independent functional status and no comorbidities that might prolong the hospital stay. Apart from this criterion, the patient is thoroughly assessed by a medical consultant with proper documentation. The medical team conducts huddles four times daily to ensure that effective communication is maintained for admission to the AMU, along with a high standard of care and discharge planning for all patients, thus allowing a good flow of patients from the ED.

AMU at HGH has evolved since its creation and has become the central hub for daily activity in the medical department. Apart from a referral from the ED, elective admission for routine procedures like renal or plural biopsy and assessment for an ambulatory condition like transient ischemic attack (TIA) are made. In the previous model before the AMU, these patients would attend ED for admission, which was a massive burden on the ED. Apart from clinical care, AMU has a dedicated educational curriculum based on improving patient care, medical knowledge, procedure, and communication skills and professionalism. Daily morning huddle round with a consultant 7 days a week is the backbone of the teaching curriculum.

The availability of bedside ultrasound as a part of the point-of-care ultrasound training program is helping the trainee residents to improve their procedural skills. Trainees are encouraged to participate in quality and audit projects with support from the residency quality program. Apart from this, there are adequate opportunities to improve clinical examination and communication skills for the Arab Board and Practical Assessment of Clinical Examination Skills (U.K.) membership examinations.

We aim to report an analysis of the AMU system in Qatar for the first time to provide insight into its effects on acute patient care and management.

## Material and Methods

### Study Population

Retrospective data from the AMU unit at HGH, Qatar, were analyzed from January 2019 through December 2020 to study the effectiveness of the AMU model of care. The criteria of effectiveness are based on the LOS and the number of readmissions. All patients admitted to our health care system are enrolled in the CERNER, a cloud-based electronic health record software used by health organizations of various sizes and specialties to streamline their operations and provide outstanding health care. It offers charting, documentation, review management, and health analytics.

### Structure and Workflow of AMAU

A dedicated 35-bed AMU was established in June 2015 at HGH to look after acute medical patients with an anticipated LOS of less than 72 hours. The AMU has a medical team led by a consultant and is supported by a MDT consisting of senior nursing staff, case managers, discharge coordinators, clinical pharmacists, social workers, physiotherapists, and occupational therapists. AMU has its own point-of-care testing facilities, including glucometers, ketometers, urine analyzer, arterial blood gas machine and ultrasound, 4 beds with a central cardiac monitoring facility, and a MDT meeting room. Blood investigations, imaging, and endoscopy requests are prioritized for patients in the AMU wherever possible. The patient is thoroughly assessed by a medical consultant with proper documentation. The medical team conducts huddles four times daily to ensure that effective communication is maintained for admission to the AMU, along with a high standard of care and discharge planning for all patients, thus allowing a good flow of patients from the ED.

### Follow-Up and Outcome

The AMU is supported by a designed ambulatory care area, where discharged patients are followed up as per need assessment. Almost all patients are seen within 1 week of discharge unless a later follow-up is desirable.

### Statistical Analysis

The data was analyzed for demographic characteristics of the patients, LOS, readmission rate, and postdischarge follow-up. A literature review was also performed for the results from other AMU facilities outside Qatar to compare with our results.

## Results

**Fig. 1:**
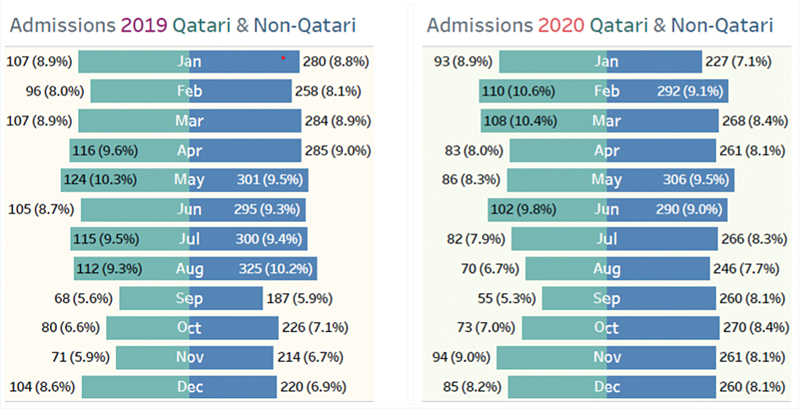
Total number of admission under acute medical unit (AMU) during 2019 (left side)–2020 (right side), light blue bar showing national and dark blue bar nonnational patients, respectively.

**Fig. 2:**
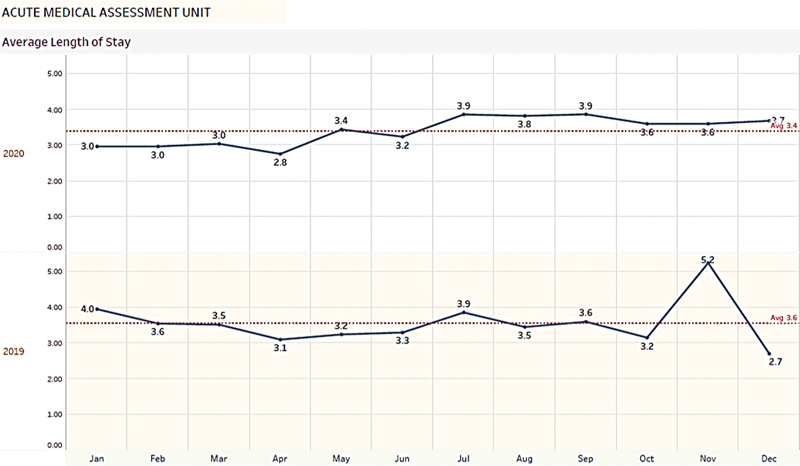
Average length of stay in acute medical unit (AMU) during 2019 and 2020.

A total of 8,371 patients were admitted under AMU from January 2019 to December 2020. Of them, 2,246 (26%) were nationals, while 6,125 (73.16%) were nonnational residents, as shown in Fig. 1 . Nonnational residents were mainly from Bangladesh, India, Egypt, the Philippines, and Nepal. The average LOS was approximately 3.2 days (2.7% in 2019 and 3.7% in 2020) ( Fig. 2 ). About 88.7% of the patients were discharged to home, 7.8% were transferred to medical wards due to longer stay, and 0.5% of the patients left the hospital against medical advice. The overall mortality rate was 0.5% ( Table 1 ).

**Table 1:** Analysis of data of discharge disposition ( *N* = 8,371)

Discharge disposition	Number (%)
Discharge to home	7,424 (88.7)
Deployed to medical wards	649 (7.8)
Discharge against medical advice	45 (0.5)
Discharge to another hospital	200 (2.4)
Absconded	8 (0.1)
Death	45 (0.5)
Total	8,371 (100)

**Fig. 3:**
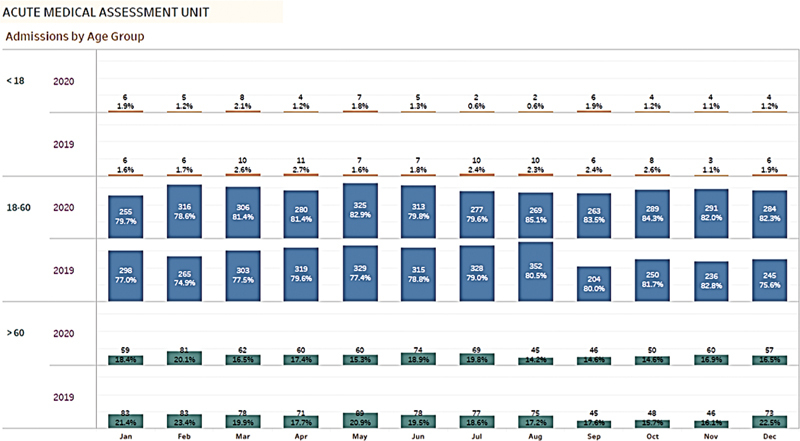
Admissions in acute medical unit (AMU) based on age groups.

The age range of the patients admitted to the AMU was 18 to 60 years, as shown in Fig. 3 . The top primary diagnoses of the patients in the AMU unit were minor stroke, TIA, chest infection (mild to moderate severity), urinary tract infection, and gastrointestinal and liver disorders including acute pancreatitis, inflammatory bowel disease, gastroenteritis, ascites, and hepatic encephalopathy. The most common comorbidities associated with patients admitted to the AMU were hypertension, diabetes mellitus with and without complications, acute kidney injury, and chronic kidney disease.

**Fig. 4:**
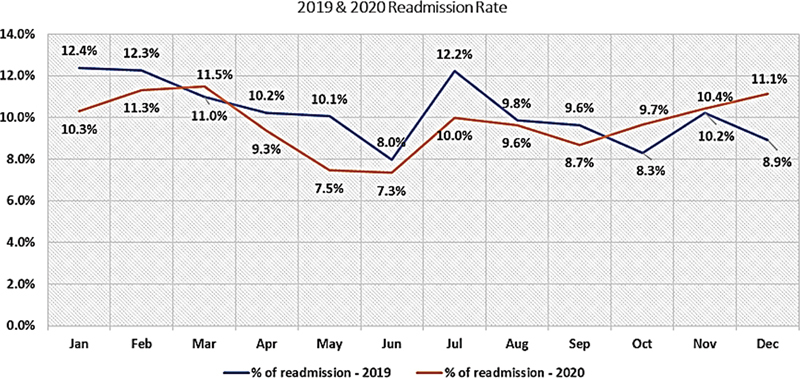
Total number of patients readmitted in acute medical unit (AMU) during 2019 (blue line) and 2020 (red line).

Analysis of 28-day readmission in the AMU has shown an overall rate of 10.25 and 9.9% in 2019 and 2020, respectively, as shown in Fig. 4 . A total of 2,858 patients were booked for a follow-up visit in the postdischarge clinic on discharge from the AMU (1,405 patients in 2019 and 1,453 patients in 2020, respectively). Table 2 shows the analysis of postdischarge clinic data in the two studied years.

**Table 2:** Analysis of patients' follow-up data in the postdischarge clinic during years 2019 and 2020

Discharge disposition	Year 2019 Number (%)	Year 2020 Number (%)	Total Number (%)
Total booked patients	1,405	1,453	2,858
- Average of booked patients/month	117	122	−
Reported patients to the clinic	1,145 (81.5)	1,342 (92.4)	2,487 (87)
- Average of reported patients/month	98	112	−
No show rate	260 (18.5)	111 (7.6)	371 (13)
- Average of no show/month	22	9	−
Discharge after first visit	942 (67)	1,145 (78.8)	2,087 (73)
Referral to the outpatient clinics for follow-up	203 (14.4)	197 (13.5)	400 (14)
Readmission rate	15 (1.1)	13 (0.9)	28 (1)

## Discussion

Our study is the first analysis of our hospital's AMU model of care, providing helpful insight into its effects on patient care and management. This model of care has proven beneficial for the smooth disposition of patients from the ED with reduced LOS, readmission rate, and overall mortality. 4 7 The average LOS in our AMU is 3.2 days, with 88.7% of patients discharged to home. The mortality rate is 0.5%, and the 28-day readmission rate is approximately 10.07%. Similar models of care in other countries have seen a reduction in patient load in the ED with smooth disposition, reduced length of hospital stay, and subsequent better hospital resource utilization with an overall mortality benefit. 4 8 9 10 11 12 13 14 15 Conway et al have also reported a decrease in ED waiting for numbers by 43% with an established AMU system. 16 The proven efficacy of AMU can be attributed to focused care with designated nursing staff and medical teams under the supervision of a medicine consultant. 9 12 13 17 18

Our results are comparable to reported results in the literature regarding total LOS, readmissions, and overall mortality benefit. Goh et al and Reid et al have reported reduced mortality, a reduction in 28-day readmissions, and an association with an increased proportion of patients discharged on the day they were admitted in the presence of a medicine consultant. 7 12 Similarly, McNeill et al reported a mean 1.3-day reduction (95% confidence interval 0.01–2.67) in LOS and a 9.2% increase in same-day discharge from the hospital due to consultant-led service in AMU. 19

Conway et al and Suthers et al reported a decrease in median hospital LOS from 7.1 to 6.9 days and 6.8 to 5.2 days, respectively, after the establishment of the AMU model of acute care. 20 21 Beckett et al reported a reduction in LOS and 30-day mortality rate of AMU patients where dedicated staff looks after only AMU patients and a consultant is available for advice. 22

Studies have shown that procedural deficiencies, including waiting for investigations and input from other specialties, contribute to increased LOS, which impacts patient flow. We established pathways for elective admission for daycare procedures like renal, pleural, or lymph node biopsy and therapeutic ascitic drainage and lumbar punctures in our AMU, which proved very effective in reducing the burden on ED and inpatient medical wards.

Identifying discharge barriers and needs by the MDT is crucial in timely discharge from the hospital. 23 Multiple daily MDT huddles can best address discharge delays as clear communication on interventions that address patient-specific needs among all health care professionals is ensured. 20 21 22 23 24 MDT-based discharge planning with close follow-up in the AMU clinic in our AMU has helped us maintain a 28-day readmission rate of around 10%, comparable to that of other AMUs internationally, which is between 8 and 28%. 9 12 22 One report from a Scottish district hospital where rapid access AMU medical clinic has been introduced has reported a statistically nonsignificant 4% decrease in readmissions and a statistically significant 9% increase in the proportion of patients discharged on admission day. 25

Our study is observational and limited to the data primarily collected for administrative purposes and may not represent the whole spectrum of clinical illnesses. We also have yet to explore patient and staff satisfaction using the AMU model of care. However, it is strengthened by the comprehensive record of all patients who present to our health care system. Furthermore, our study represents the first observation of the AMU system in the Middle East region and adds to the growing body of literature supporting the spread of this unique health care delivery model.

## Conclusion

Worldwide health care provision has become a challenge due to the growing number of patient's chronic medical problems and the aging population. It is also associated with a decrease in many acute care beds and an increase in bed crisis for the required admissions, causing crowding of ED with more than 85 to 90% of bed occupancy. 8 The results of patient care and management under the AMU facility at HGH, Qatar, are promising and signify the effectiveness of this system. The primary factor responsible for the efficacy of the AMU services is a dedicated, well-equipped department with dedicated nursing staff and a medical team under the supervision of a medical consultant. Other factors are consultant-led and delivered services, a dedicated MDT team, dedicated bed coordinators, and a postdischarge follow-up clinic. Further studies are needed to establish the effectiveness of AMU and health care team, patient, and family satisfaction.
